# Calcified, minimally Fat-contained angiomyolipoma clinically indistinguishable from a renal cell carcinoma

**DOI:** 10.1186/1471-2369-14-160

**Published:** 2013-07-22

**Authors:** Chin-Li Chen, Shou-Hung Tang, Sheng-Tang Wu, En Meng, Chih-Wei Tsao, Guang-Huan Sun, Dah-Shyong Yu, Sun-Yran Chang, Tai-Lung Cha

**Affiliations:** 1 Division of Urology, Department of Surgery, Tri-Service General Hospital, No.325, Section 2, Cheng-Kung Road, Taipei 114, Taiwan, R.O.C

**Keywords:** Angiomyolipoma, Calcifications, Radical nephrectomy, Renal cell carcinoma

## Abstract

**Background:**

Angiomyolipomas are benign tumors of the kidney. Typical angiomyolipomas are usually recognized by identifying fat components before any intervention. On the contrary, solid renal masses without evident fatty components but containing calcifications on the computed tomography scan are suspicious for malignancy. However, as in this rare case, rules of diagnostic imaging are of exceptions.

**Case presentation:**

A 40-year-old man presented with left flank pain. The plain X-ray showed multiple coarse calcifications of 4.0 x 3.2 cm in diameter on the left upper quadrant abdomen. Computed tomography scan further revealed a solid renal mass and inside the mass there were calcifications. The size of the tumor was 5.6 × 5.5 × 6.3 cm. We performed a radical nephrectomy, and the histopathology showed a minimally fat-contained angiomyolipoma of multiple calcifications. The patient was free of recurrence or metastases after a follow-up period of 3 years.

**Conclusion:**

An angiomyolipoma containing calcification is rare. An angiomyolipoma with minimal fat concomitant with calcifications is an even rarer presentation. It is very difficult to differentiate a minimal-fat angiomyolipoma with calcifications from a renal cell carcinoma preoperatively. In such a circumstance, a well-planned partial nephrectomy may be optimal for the patient, regardless of the tumor size.

## Background

Angiomyolipomas (AMLs) of the kidney can be diagnosed mostly based on intratumoral fat components on computed tomography (CT) examinations [1]. AMLs containing calcification are rare and only four cases were reported in literature. In the reported cases, fat-contained tumors were generally identified by imaging [2-5]. Several studies have addressed the importance of calcifications inside a solid renal mass. Briefly, about 40% of calcified solid renal mass will be malignant, and neither the number nor the pattern of calcifications predicted malignancy [6]. Therefore, it is challenge to differentiate an AML from a renal cell carcinoma (RCC) when the CT examinations show calcifications, especially when fatty components are not identified by imaging. To our knowledge, calcified AMLs without fat component have not been reported.

## Case presentation

A 40-year-old man presented with intermittent left flank pain for one month. He denied having fever or any voiding symptoms. The physical examination was unremarkable. There was no weight loss, night sweat, or other constitutional symptoms.

**Figure 1:**
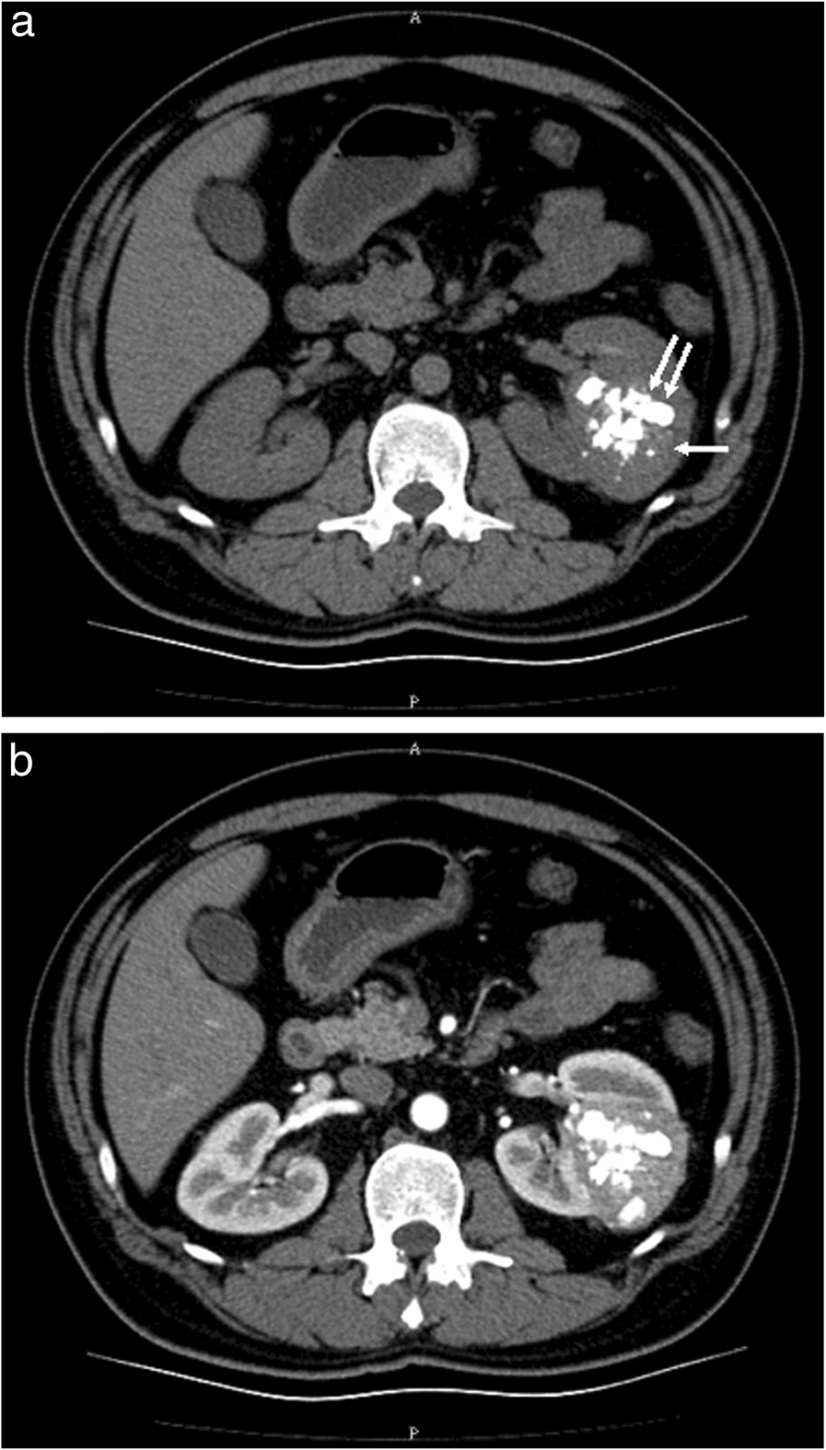
**CT scan of the angiomyolipoma (AML) of the left kidney. (a)** Noncontrast scan showing a solid tumor (arrow) with multiple calcifications (double arrow). **(b)** Contrast CT imaging showed contrast enhancement.

A plan X-ray showed multiple amorphous calcifications in the patient’s left upper abdomen; the calcifications were 4.0 × 3.2 cm in diameter on the left upper quadrant abdomen. Intravenous urography (IVU) disclosed a mass-like defect with multiple calcifications in the middle of the left kidney but without any obstruction. Incomplete ureteral duplication was also found incidentally in the right kidney. CT scans further revealed a 5.6 × 5.5 × 6.3 cm, relatively well-circumscribed, heterogeneous, bulging mass lesion with same coarse calcifications in the middle of the left kidney (Figure 1). The density of the lesion measured about 51 Hounsfield units (HU) on non-enhanced images. After contrast enhancement, the CT density rose up to 125 HU. On magnetic resonance imaging (MRI), the mass showed intermediate T1 signals and intermediate-to-low T2 signals, but no detectable fat component could be identified on either fat-suppressed or chemical-shifted images The preoperative diagnosis of this renal mass was a RCC.

**Figure 2:**
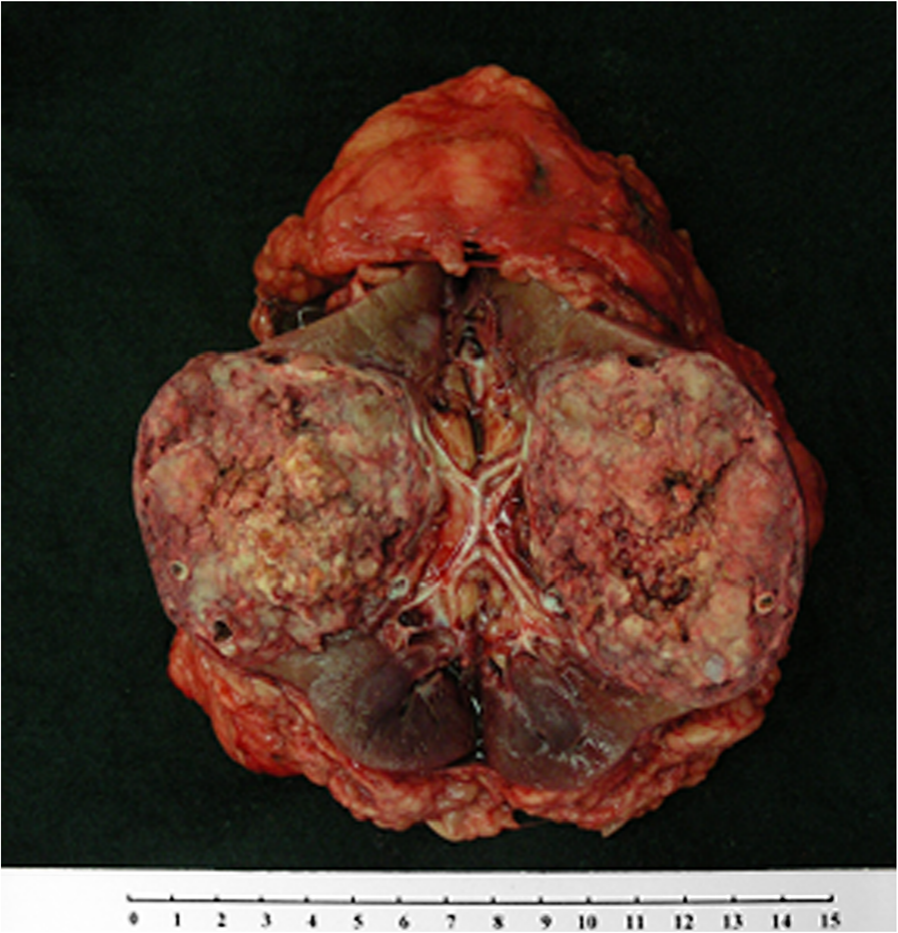
Grossly the minimal-fat AML showed a capsulated solid mass with multiple calcifications.

**Figure 3:**
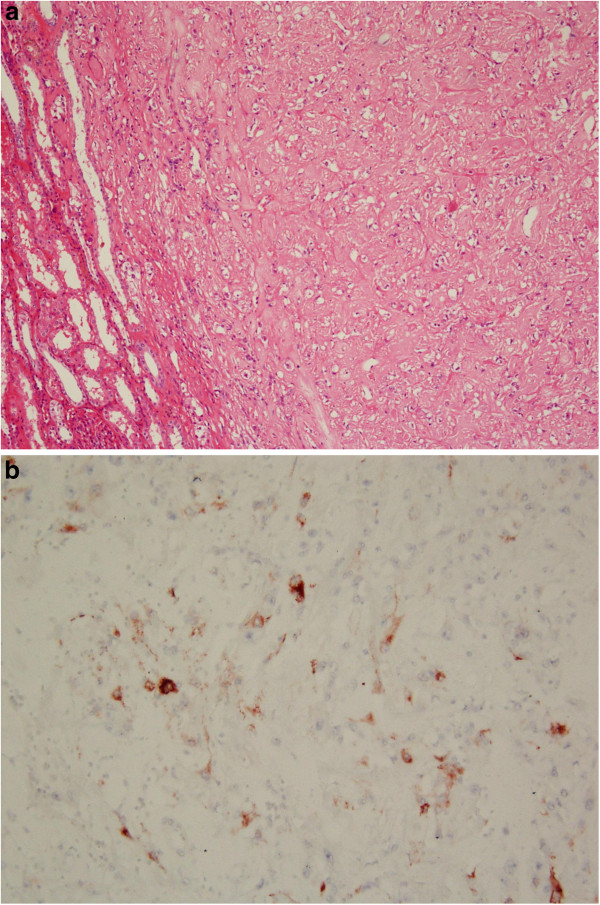
**Hematoxylin-eosin stain and immunohistochemical stain of the tumor. (a)** Hematoxylin-eosin stained photomicrograph with 100× magnification demonstrating epithelioid cells. **(b)** photomicrograph of the epithelioid angiomyolipoma showed positive staining with the HMB-45 immunohistochemical stain with 200× magnification.

We therefore performed a transperitoneal left radical nephrectomy. Grossly it was an encapsulated solid tumor measuring 6.0 × 6.0 × 5.2 cm mixed with reddish and yellowish material (Figure 2). Histopathology, it showed extensive hyalinization intermixed with marked calcification, focal smooth muscle cells, small vessels, adipocytes, and short spindle perivascular epithelioid cells, and was compatible with an angiomyolipoma. There were 30% epithelioid cells and 0 mitotic figures per 10 high-power fields. No atypical mitotic figures or necrosis was seen (Figure 3a). The hyalinization was evident on Masson stain, and immunohistochemical stains showed diffusely positive for actin and HMB-45 in the tumor (Figure 3b). There was neither recurrence, nor distant metastases at 3rd year of postoperative follow-up.

An AML is a benign tumor lesion of the kidney composed of adipose tissue, smooth muscle and thick-walled blood vessels [1]. The incidence of AML is approximately 0.3% [7]. It is important to differentiate an AML from a RCC because observation and regular follow-ups can be appropriate for patients with asymptomatic small AMLs. By contrast, surgical resection is generally necessary for a RCC [8].

AMLs contain a variable proportion of fat as the leading presentation on CT examination and this is the most important characteristic for diagnosis [3]. On T1-weighted MRI images, AMLs typically present with high signal intensity along with marked fat-suppressed regions of decreased intensity [1]. In most cases of AMLs, CT examination can provide an adequate diagnosis base on this characteristic of a fatty component. AMLs account for 5.7% of renal masses suspected of being a RCC preoperatively and subjected to nephrectomy [9]. However, minimal-fat AMLs are difficult to differentiate from RCCs because of the absence of fat or undetectable fat contents on CT imaging [8].

Renal epithelioid angiomyolipomas (EAMLs), also known as epithelioid PEComas, were distinct variants of AMLs, and have been reported to be less fat-contained, and more aggressive than the other common AMLs. In a report by Froemming and colleagues, 6 out of 9 cases of EAMLs had small amount of fat seen by preoperative imaging. They concluded that EAMLs can be indistinguishable from a RCC preoperatively [10,11].

There are only four case reports on AMLs with calcification [2-5]. RCCs are highly suspected when encountering solid renal tumors without fatty components but with multiple calcifications. However, it has been reported that RCCs, in particular, papillay RCCs, may present with fat-contained tumors [12].

Merran et al. suggested that when the fat components fill most of the renal tumor, an AML is the more likely diagnosis; on the contrary when the predominant component is calcification, a RCC is more likely than an AML [3]. Diffusion-weighted MRI scans show homogeneous diffusion in minimal-fat AMLs. On the contrary, clear cell RCCs present with heterogeneous diffusion imaging [8].

The unique nature of this case is that a preoperative CT examination showed a heterogeneously contrast-enhancing mass lesion occupying the left kidney with obvious calcification. The calcifications may be the result of previous hemorrhage, but this is only our speculation. We also did IVU and MRI to evaluate the possibility of renal stones or other renal malignancy. However, these examinations did not show other significant findings.

## Conclusion

An AML containing small amount of fatty components along with multiple coarse calcifications is extremely rare. It is very challenge to differentiate minimal-fat AMLs from RCCs preoperatively, as is seen in our case herein. With this radiological pattern, epitheloid AMLs should be included in the differential diagnosis in the future.

## Consent

Written informed consent was obtained from the patient for publication of this case report and any accompanying images. A copy of the written consent is available for review by the Editor of this journal.

## Competing interests

The authors declare that they have no competing interest.

## Authors’ contributions

All authors participated in the patient’s diagnosis and medical management. Chen JL drafted the first version of the manuscript and Cha TL drafted the revised manuscript. All authors have read and approved the final manuscript.

## Pre-publication history

The pre-publication history for this paper can be accessed here:

http://www.biomedcentral.com/1471-2369/14/160/prepub
